# New Constitutive Model for the Size Effect on Flow Stress Based on the Energy Conservation Law

**DOI:** 10.3390/ma13112617

**Published:** 2020-06-08

**Authors:** Chuanjie Wang, Haiyang Wang, Gang Chen, Qiang Zhu, Lingjiang Cui, Peng Zhang, Anping Dong

**Affiliations:** 1 School of Materials Science and Engineering, Harbin Institute of Technology at Weihai, Weihai 264209, China; cjwang@hitwh.edu.cn (C.W.); 18s030154@stu.hit.edu.cn (H.W.); cg@hitwh.edu.cn (G.C.); zhuqiang@hit.edu.cn (Q.Z.); cuilj@hitwh.edu.cn (L.C.); 2 Key Laboratory of Micro-Systems and Micro-Structures Manufacturing of Ministry of Education, Harbin Institute of Technology, Harbin 150080, China; 3 Shanghai Key Laboratory of Advanced High-temperature Materials and Precision Forming, Shanghai Jiao Tong University, Shanghai 200240, China

**Keywords:** energy conservation law, constitutive model, deformation behavior, size effect

## Abstract

In this study, a new model involving energy is established to characterize the size effect on flow stress. The new model treats the experimental machine and the specimen as an isolated system, and this isolated system satisfies the Energy Conservation Law. The total work performed on the specimen by the experimental machine is nearly equal to the energy consumed by the specimen plastic deformation and the energy consumed by friction (which can be ignored when working without friction). The new model predicts the energy consumption of the specimen deformation by quantifying the total energy input to the specimen by the experimental machine and then obtaining the relevant parameters of the constitutive model. Through uniaxial tensile tests of pure nickel thin sheets with various thickness/average grain sizes (*t/d*), the new model was used to optimize the parameters of the existing constitutive model that predicts the flow stress of specimens with different *t/d*. The prediction accuracy of the optimized constitutive model is improved, especially for specimens with a *t/d* < 1. The new model is established from the perspective of energy input to avoid the analysis of the material deformation mechanism and improve the prediction accuracy.

## 1. Introduction 

Theoretical and experimental research on the size effect of metal sheets is stimulated by the widespread use of micro-components [1]. As an unavoidable challenge in microforming, the size effect has received extraordinary attention from researchers in the plastic-forming field [2,3]. Since the innovative discovery of Geiger [4] and Engel [5], experimental characterization and tissue analysis have made great progress and several constitutive models have been proposed in experimental observations. The Hall-Petch relationship, which describes the relationship between flow stress and grain size, is the most frequently modified to describe the size effect. Based on the experimental observations, the feature size coefficient thickness/average grain size (*t/d*), describing the grain number in the thickness direction, was added to the traditional constitutive equation to fit the experimental results [6,7,8]. However, the applicable range of the *t/d* ratio is only between 1 < *t/d* < (*t/d*)_c_ (critical value) [9,10]. (*t/d*)_c_ is the critical value of the size effect on the flow stress of the thin sheet. When the *t/d* of the sheet drops to (*t/d*)_c_, the flow stress of the thin sheet significantly decreases and deviates from the Hall–Petch relationship [11,12]. Not only the flow stress but also the deformation behavior [13] and fracture behavior [14] of the thin sheet also change significantly, and this is the so-called size effect phenomenon. To further express the fundamental deformation mechanism of the size effect, dislocation density models were constructed to describe the variation in dislocation density in the surface grain and in inner grain [15,16,17,18]. According to these findings, Geiger [4] and Engel et al. [5] established a surface layer model that can effectively describe the size effect and was widely applicable for investigators. Based on the surface layer model, many new constitutive models combining different models were exploited to represent the size effect on the flow stress [2,8,19,20,21,22]. The surface layer model has been studied extensively and in-depth, but is only applicable when the *t/d* > 2 [9]. The deformation behavior of specimens with only one grain in the thickness direction has been initially studied [23,24]. According to the above analysis, the constitutive models based on the microstructure and deformation behavior has limitations. 

The foundation of these above models is the deformation mechanism of the material. Because all factors of the mechanism of deformation behavior cannot be considered comprehensively, the existing constitutive model deviates from the experimental results in certain conditions. However, what if we establish a corresponding model from the input side? This model can describe the variation in the output (deformation behavior) by predicting the energy at the input. According to the Energy Conservation Law, the total energy remains unchanged after energy conversion in an isolated system. If the experimental machine and the specimen are considered as an isolated system, then this system obeys the Energy Conservation Law. The energy input by the experimental machine to the specimen is equal to the energy consumed by the specimen deformation. In terms of energy input, a new model capable of quantifying and predicting the input energy was built to describe the energy consumed by material deformation. It avoids tedious mechanism analysis and formula derivation to achieve the prediction of deformation results. Finding a model of the energy input that can effectively describe the deformation behavior has important practical significance for metal forming.

In this paper, based on the Energy Conservation Law a new model involving energy is established to characterize the deformation behavior and size effect. The new model is initially applied to modify the parameters of the flow stress constitutive equation about the size effect and achieves satisfactory results.

## 2. Application of Energy Conservation Law in Metal Forming

The Energy Conservation Law is expressed as: "The total energy of an isolated system remains unchanged." The experimental machine and specimen are considered an isolated system. The mechanical energy generated by the experimental machine is converted into the internal energy-consumed deformation in this isolated system for most cold-working metal forming (drawing, bulging, tension, compression, etc.). In energy conversion, the energy consumed by friction is negligible for proper lubrication conditions. The application of the Energy Conservation Law in metal forming is written as:

(1) $$E_{M}=E_{I}+E_{F}$$

where *E*_M_ is the mechanical energy produced by the experimental machine, *E*_I_ is the internal energy consumed by the specimen deformation and *E*_F_ is the energy lost by friction during forming.

## 3. New Model Based on Energy Conservation

### 3.1. Model Establishment

The plastic deformation must be accompanied by energy conversion, which is the conversion of the mechanical energy of the experimental machine into the internal energy of the specimen. Taking the quasi-static uniaxial tensile tests as an example, the essence of the uniaxial tensile tests is a process in which the specimen gauge generates a certain displacement under tension. According to Equation (1), the mechanical energy exerted by the experimental machine on the specimen is equal to the internal energy consumed by the specimen deformation. The internal energy absorbed by the deformation is distributed through two fields of grain deformation (slipping and twining) and thermal energy dissipation. When there is no thermal energy input during the deformation process and the strain rate is quasi-static, thermal energy accounts for a small proportion and can be ignored. Therefore, most of the internal energy consumed by the deformation contributes to grain deformation, which can be reflected in the true stress-strain curves. Additionally, the mechanical energy exerted by the experimental machine is reflected in the force-displacement curves. The physical meaning of the integral of the force-displacement curve is the total work done by the experimental machine, but the physical meaning of the integral of the true stress-strain curve is unclear. There must be a specific relationship between both sides according to the Energy Conservation Law, as shown in Equation (2).

(2) $$\int_{0}^{\Delta l_{a}}Fd\Delta l\propto \int_{0}^{\varepsilon_{a}}\sigma_{t}(\varepsilon_{t})d\varepsilon_{t}$$

where *F* is the tensile force, Δ*l* is the displacement of the specimen gauge, Δ*l*_a_ is the displacement at any time, *σ*_t_ is the true stress, *ε*_t_ is the true strain and *ε*_a_ is the true strain at any time.

As we all know, the true stress-strain curve is obtained by converting the engineering stress-strain curve, as shown in Equation (3).

(3) $$\sigma_{t}=\sigma (1+\varepsilon )\;\mathrm{and}\;\varepsilon_{t}=ln(1+\varepsilon )$$

where *σ* is the engineering stress and *ε* is the engineering strain.

(4) $$\sigma =\frac{F}{S_{0}}\;\mathrm{and}\;\varepsilon =\frac{\Delta l}{l_{0}}$$

where *S*_0_ is the initial sectional area of the gauge and *l*_0_ is the initial gauge length.

When Δ*l* = Δ*l_a_*, *ε_t_* = ln(1 + *ε*) = ln(1 + $\frac{\Delta l}{l_{0}}$) = ln ln(1 + $\frac{\Delta l_{a}}{l_{0}}$) = *ε_a_*.

According Equations (3) and (4), $\int_{0}^{\varepsilon_{a}}\sigma_{t}(\varepsilon_{t})d\varepsilon_{t}$ can be written as the following:

(5) $$\int_{0}^{\varepsilon_{a}}\sigma_{t}(\varepsilon_{t})d\varepsilon_{t}=\int_{0}^{\varepsilon_{a}}\sigma (1+\varepsilon )d\ln(1+\varepsilon )=\int_{0}^{\varepsilon_{a}}\frac{F}{S_{0}}(1+\frac{\Delta l}{l_{0}})d\ln(1+\frac{\Delta l}{l_{0}})=\frac{1}{S_{0}l_{0}}\int_{0}^{\Delta l_{a}}Fd\Delta l=\bar{E}$$

$\bar{E}$ indicates the energy consumption per unit volume of the deformation zone through plastic deformation. Equation (5) describes that the integral of the true stress-strain curve is equal to the ratio of the integral of the force-displacement curve to the gauge volume. In addition, we can get that $\sigma_{t}(\varepsilon_{t})↔\frac{F}{S_{0}l_{0}}$. This indicates that the true stress-strain curve relates to the force on the specimen per unit volume. Additionally, the force of the specimen per unit volume reflects the stress state of the specimen per unit volume. Due to the inhomogeneity of the properties of the specimen per unit volume, the true stress-strain curve reflects the average stress state of the specimen per unit volume in the deformation region. The stress state of a material during deformation depends fundamentally on the microstructure. Grain size is a key parameter that affects material properties, while the grain size determines the number of grains per volume. Therefore, the true stress-strain curve is closely related to the grain number per unit volume (N).

**Figure 1:**
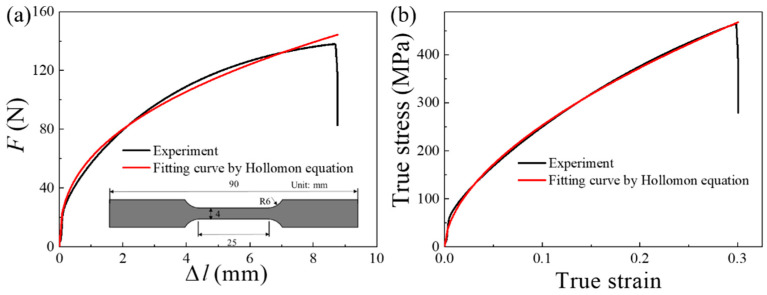
(**a**) Force-displacement curve (**b**) True stress-strain curve of the same specimen.

To verify the correctness of Equation (5), one of the random experimental results from the uniaxial tension tests on pure Ni sheets is selected to be described. Firstly, the force-displacement curve and the corresponding true stress-strain curve are fitted by the Hollomon equation [25] ($\sigma =K\varepsilon^{n}$) to obtain the functions of *K* and *n* values, as shown in Figure 1 and Equation (6). Then, the area formed by these two curves and the X axis is obtained using a definite integral, as shown in Figure 1 and Equation (7). The volume of the specimen gauge is $S_{0}l_{0}=t_{0}\times b_{0}\times l_{0}=0.1\times 4\times 25=10\;{\mathrm{mm}}^{3}=1\times 10^{-8}\;m^{3}$, where *t*_0_ is the initial specimen thickness, *b*_0_ is the initial width of the gauge section and *l*_0_ is the initial length of the gauge section. The results of the calculations according to Equations (6) and (7) can indicate that Equation (8) is proven. Three specimens with different geometric sizes were selected and the above steps are repeated. All the results can prove the correctness of Equation (5).

(6) $$F=60.7\Delta l^{0.4}(10^{-3}J)\;\mathrm{and}\;\sigma_{t}=915.9\varepsilon_{t}{}^{0.56}(10^{6}J)$$

(7) $$\left\{ \begin{array}{l} {\int_{0}^{\Delta l_{a}}Fd\Delta l=\int_{0}^{8.8}60.7\Delta l}^{0.4}d\Delta l=910.6\times 10^{-3}J\\ {\int_{0}^{\varepsilon_{a}}\sigma_{t}d\varepsilon_{t}=\int_{0}^{0.3}915.9\varepsilon_{t}}^{0.55}d\varepsilon_{t}=91.4\times 10^{6}J \end{array}\right.$$

(8) $$\bar{E}=\frac{1}{S_{0}l_{0}}\int_{0}^{\Delta l_{a}}Fd\Delta l\approx \int_{0}^{\varepsilon_{a}}\sigma_{t}d\varepsilon_{t}$$

### 3.2. Verification Based on Finite Element Simulation

**Figure 2:**
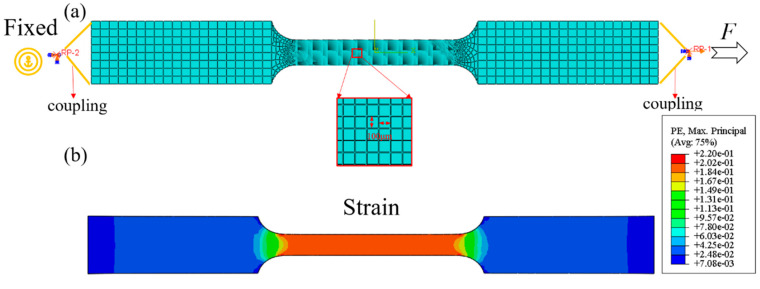
(**a**) Finite element model of the uniaxial tension test (**b**) Strain distribution.

**Figure 3:**
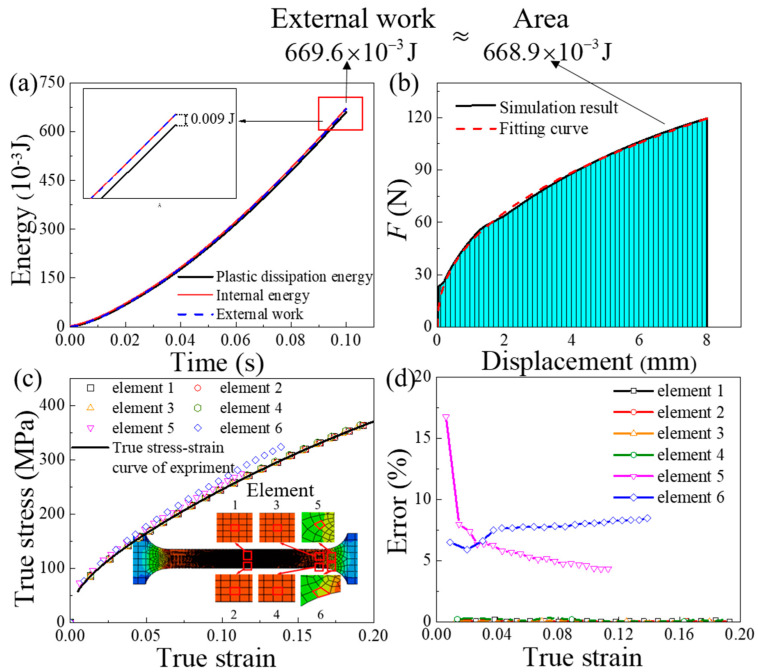
(**a**) Variation in different types of energy (**b**) Force-displacement curve of RP-1 (**c**) True stress-strain curves of the elements at different positions (**d**) Error between the experimental results and the simulation.

Finite element simulation can intuitively describe the stress and strain per unit volume of the specimen and the energy conversion of the entire system. Using the ABAQUS 6.14.1 software (Dassault Systemes, Vélizy-Villacoublay, France), a three-dimensional model of the uniaxial tension test for the thin plate was established, as shown in Figure 2a. The gauge section of the specimen was divided into 10,000 meshes with a size of 0.1 × 0.1 × 0.1 mm^3^ to represent the deformation area per unit volume. The simulation results show that the gauge area is the main deformation area, as shown in Figure 2b. In the historical output, the energy change in the entire model (including the internal energy, external work and plastic dissipation energy) and the force-displacement curve of RP-1 were the output. In the field output, the stress-strain curve of a single element and the plastic dissipation energy of a single element were outputs. The external work, internal energy change and plastic dissipation energy of the entire model are shown in Figure 3a.

It is obvious that the external work is approximately equal to the internal energy, which is slightly higher than the plastic dissipation energy. In the Abaqus software, the expression of energy conservation for the overall model can be written as:

(9) $$E_{I}-E_{W}=\mathrm{constant}\;\mathrm{and}\;E_{I}=E_{E}+E_{P},$$

where *E*_I_ is the internal energy, *E*_W_ is the external work, *E*_E_ is the recoverable elastic strain energy and *E*_P_ is the plastic dissipation energy. Figure 3b shows the force-displacement curve of the finite element simulation. The force-displacement curve is fitted to $F=48.9\Delta l^{0.43}$.

The area enclosed by the force displacement curve and the X axis can be written as:

(10) $$\int_{0}^{8}Fd\Delta l=\int_{0}^{8}48.9\Delta l^{0.43}d\Delta l=668.9\times 10^{-3}J.$$

The area enclosed by the force-displacement curve and the X-axis is approximately equal to the external work. The true stress-strain curve of several elements in the gauge part (elements 1–4) and the experimental true stress-strain curve matched well, as shown in Figure 3c. The method of calculation of the error is the ratio of the difference between the true stress of the simulation and the true stress of the experiment to the true stress of the experiment. The error between the simulated flow stress of elements 1–4 and the experimental flow stress is about 0.1% in Figure 3d. However, the true stress-strain curve of the elements in the transition part (elements 5,6) deviated significantly from the experimental results. Therefore, the true stress-strain curve of the specimen describes the true stress-strain state of the unit volume of the material in the gauge section (the main deformation area).

### 3.3. Discussion

According to the simulation results, the increased internal energy is mainly dissipated by plastic deformation. In other words, the absorbed internal energy of the specimen mainly contributes to the grain deformation (slipping and twining). With the increasing grain number per unit volume, the increasing grain boundary strengthening [26,27] and strong dislocation density [12,28] are obtained to enhance the deformation resistance, and this ultimately leads to an intensified energy consumption. Therefore, there is a certain relationship between $\bar{E}$ and the grain number per unit volume. Through experimental observation, *t/d* has a significant effect on the deformation behavior of the metal sheet [6,7,8]. Therefore, for metal sheets *t*^3^*/d*^3^ (thickness/grain size) can be regarded as the number of grains per unit volume.

(11) E¯∝t3d3.

**Figure 4:**
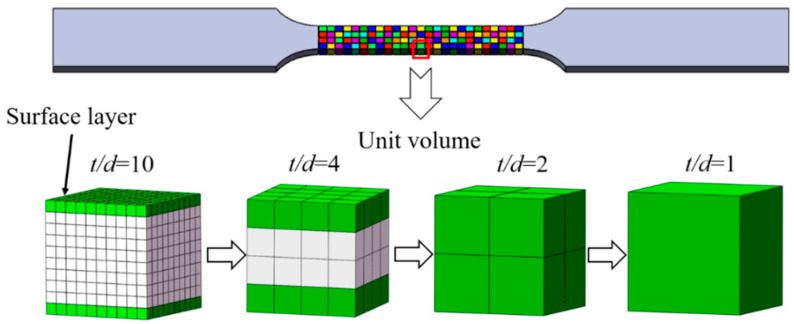
Grain number per unit volume and surface layer grains with decreasing thickness/average grain sizes *(t/d).*

The true stress-strain curve reflects the average stress-strain state of the grains per unit volume (N). The model of the grains per unit volume is shown in Figure 4. As the number of grains per unit volume decreases, the energy required for deformation decreases. Combined with the surface layer model, the new model can excellently characterize the size effect of metal sheets. According to the surface layer model, the sheet is divided into surface layer grains and internal grains, and the material surface cannot hinder the movement of dislocations like grain boundaries [4]. The movement of dislocations in the surface grains is not limited by the same internal grains, resulting in less hardening [19]. Therefore, the surface grains suffer from a lower flow stress compared to the inner grains. As the specimen size decreases, the proportion of surface grains increases, which reduces the flow stress of the entire specimen [29].

Macro-scale (*t/d* > *φ*_c_: critical value): *φ*_c_ is the *t/d* critical value at which thin sheets exhibit size effects. When *t/d* > *φ*_c_ (N > *φ*_c_^3^), the surface layer accounts for a small proportion (N_surf_/N_total_ < 2/*φ*_c_ (grain number of the surface layer/total grain number per unit volume)). The number of grains per unit volume is high, and the number of internal grains is much higher than the number of surface layer grains; the weakening effect of the surface layer grains can be ignored. In this case, the decrease in flow stress as the grain size increases is due to a decrease in the overall dislocation density of the material affected by the increased grain size [30]. The laws of flow stress and grain size satisfy the Hall–Petch relationship [31]. The decreasing grain number per unit volume leads to a reduction in the grain boundary density [25] and intergranular back stress [5]. The decreasing grain number per unit volume is the main impetus for the reduction in flow stress.

Micro-scale (*t/d* < *φ*_c_): when the *t/d* value reaches the critical value *φ*_c_, the proportion of surface layer grains reaches a certain critical value and surface layer effect cannot be ignored [19]. The weakening of the surface layer showed significant effects as the *t/d* decreased. Thus, the flow stress of the material is significantly reduced, resulting in a deviation in the flow stress from the initial Hall–Petch relationship in relation to grain size [32]. In this case, the decrease in the number of grains per unit volume increases the proportion of surface layer grains and enhances the weakening effect of the surface layer grains. Therefore, the flow stress decreases significantly. Particularly when the *t/d* < *2*, the surface layer model is no longer applicable. The orientation and deformation behavior of individual grains is particularly important due to the weak intergranular constraint. The specimen thickness [33] and grain orientation [34] are the key factors affecting the flow stress. In this case, the flow stress of the sheet is more difficult to predict due to the different orientation of individual grains.

Additionally, the non-uniform grain size makes a difference in the number of grains per unit volume at different locations. The true stress-strain curve reflects the mean stress state of the grains in each unit volume.

### 3.4. Application

One of the applications of the new model is to optimize the parameters of the existing constitutive equations.

#### 3.4.1. Existing Flow Stress Constitutive Model

**Figure 5:**
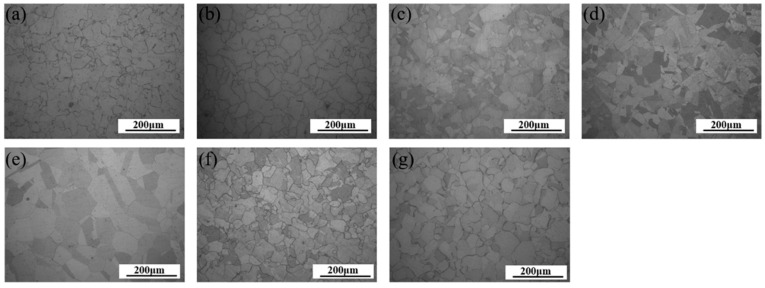
Microstructures of Ni sheets with annealing temperature of 100 μm: (**a**) 750 °C, (**b**) 850 °C, (**c**) 950 °C, (**d**) 1050 °C, (**e**) 1150 °C; 200 μm: (**f**) 750 °C, (**g**) 850 °C.

**Figure 6:**
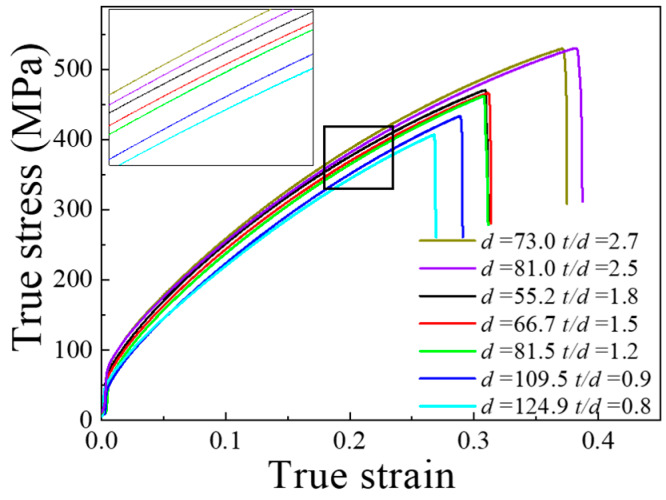
True stress–true strain curves of specimens with various *t/d.*

Uniaxial tensile tests of pure Ni with a thickness of 100 and 200 μm were implemented. The geometric size of the specimens for the uniaxial tensile experiment is shown in Figure 1a. In this paper, the specimens for the uniaxial tensile tests were prepared along the rolling direction. To obtain specimens with different grain sizes, the specimens with a thickness of 100 μm were annealed at 750, 850, 950, 1050 and 1150 °C for one hour, and the specimens with a thickness of 200 μm were annealed at 750 and 850 °C for one hour and then cooled to room temperature. The microstructures of specimens with various grain sizes are shown in Figure 5. In Figure 5, the grain shape is a hexahedron, and the grains can be seen as isometric grains. The average grain sizes were measured by the intercept method. The average grain size in Figure 5a–f is shown in Table 1 (1–7) in order. For specimens with different grain sizes in Figure 5, uniaxial tensile tests were implemented to obtain the corresponding true stress-strain curves, as shown in Figure 6. The experimental results were used to construct and verify the optimized constitutive model based on the new model involving energy. According to the Figure 6, the *K* and *n* values in the Swift equation fitted by the least square method are shown in Table 1.

Based on the Swift model, Wang et al. [35] established a new constitutive equation considering the size effects I. The Swift model is written as:

(12) $$\sigma_{t}=K{\left(\varepsilon_{0}+\varepsilon_{t}\right)}^{n}$$

Based on the Hall–Petch relationship, Wang et al. [35] obtained the following equation.

(13) $$\sigma \propto \sqrt{t/d}$$

Based on the internal grain boundary model and the surface layer model, Wang et al. [35] obtained the following equation.

(14) $$\sigma \propto {\left(t/d\right)}^{-1}$$

Combined with Equations (13) and (14), Wang et al. [35] believed that the intensity coefficient K is seen as a parameter affected by the size effect.

(15) $$K(t/d)=a+b\sqrt{t/d}+c{\left(t/d\right)}^{-1}$$

Therefore, the true stress-strain curve yields the following formula:

(16) $$\sigma_{t}=K(t/d){\left(\varepsilon_{0}+\varepsilon_{t}\right)}^{n}=(a+b\sqrt{t/d}+c{\left(t/d\right)}^{-1}){\left(\varepsilon_{0}+\varepsilon_{t}\right)}^{n}$$

where K(t/d) introduces the size effect factor (t/d) to describe the size effect on flow stress. They believed that n value for specimens with various t/d can be considered as an average constant.

Test numbers 1,2 and 6 was randomly selected to determine the *a*, *b* and *c* parameters in Equation (14). The initial fitting results are shown in Table 2. According Equation (16) and Table 2, the *a*, *b*, *c* parameters can be solved and the constitutive model established by Wang et al. is written as the following:

(17) $$K(t/d)=832.7+102.3\sqrt{t/d}+30.6{\left(t/d\right)}^{-1}$$

(18) $$\sigma_{t}=K(t/d){\left(\varepsilon_{0}+\varepsilon_{t}\right)}^{n}=(832.7+102.3\sqrt{t/d}+30.6{\left(t/d\right)}^{-1}){\left(\varepsilon_{0}+\varepsilon_{t}\right)}^{0.61}$$

**Figure 7:**
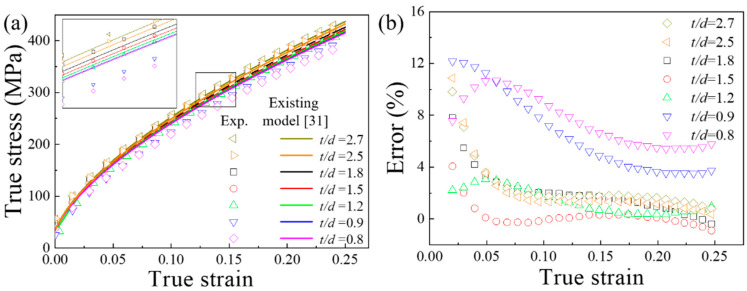
(**a**) Comparison of the experimental results and the prediction of the existing constitute model for true stress-strain curves (**b**) Errors of existing models.

Therefore, the comparison of the experimental results and Equation (18) of the true stress-strain curves are shown in Figure 7a. When *t/d* > 1, the prediction results of the constitutive model in the reference [35] were in good agreement with the experimental ones. Figure 7b shows the error between the prediction results of the existing model and the experimental results. Obviously, the error of the prediction results of the existing model for specimens with a *t/d* < 1 is significantly higher than that for specimens with *t/d* > 1. The constitutive model [35] was no longer applicable for specimens with a *t/d* < 1. The orientation and deformation behavior of individual grains have a strong influence on the flow stress for a sample with only one grain in thickness, but existing models only take into account the influence of grain size. This was the same as described in the paper of Wang et al. [35]. The method of determining the *n* value directly affects the prediction accuracy. Despite slight changes in the value of *n*, the flow stress exhibits significant fluctuations.

#### 3.4.2. Optimized Flow Stress Constitutive Model

In this paper, a new model involving energy was used to optimize *n* values for improving prediction accuracy.

**Figure 8:**
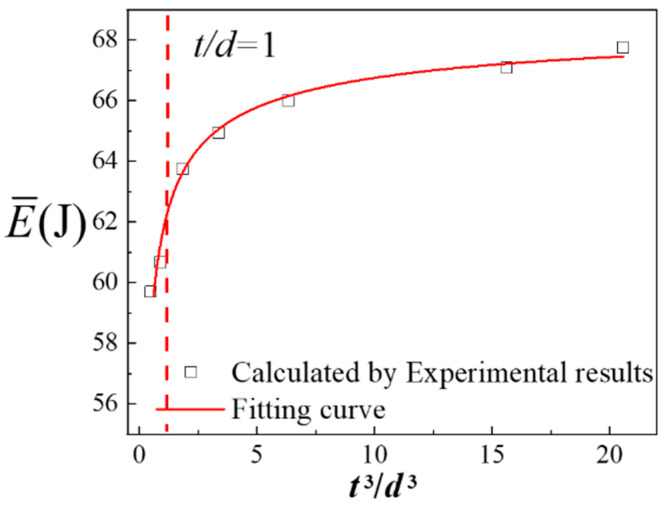
Optimized *n*_p_ value based on the new model.

Then, the value of *n* in Equation (18) is optimized by the new model involving energy. The optimized *n* value is written as *n*_p_. According the Swift equation and Table 1, the true stress-strain curves of tests 1–7 were integrated to obtain the $\bar{E}$(*ε*_t_ =0-0.25). Table 3 shows the $\bar{E}$ of specimens with various *t/d.* The data in Table 3 was fitted to the Equation (17) by the least square method, as shown in Figure 8. The function between the $\bar{E}$ and the *t*^3^/*d*^3^ is written as:

(19) $$\bar{E}=69.1-7.4{\left(\frac{t^{3}}{d^{3}}\right)}^{-0.5}\times 10^{-6}$$

Combining Equations (5), (17), and (19) can be written as follows:

(20) $$\bar{E}=\int_{\varepsilon_{1}}^{\varepsilon_{2}}\sigma_{t}d\varepsilon_{t}=\int_{\varepsilon_{1}}^{\varepsilon_{2}}K(t/d){\left(\varepsilon_{0}+\varepsilon_{t}\right)}^{n_{p}}d\varepsilon_{t}$$

(21) $$\bar{E}=\int_{0}^{0.25}(832.7+102.3\sqrt{t/d}+30.6{\left(t/d\right)}^{-1}){\left(0.0043+\varepsilon_{t}\right)}^{n_{p}}d\varepsilon_{t}\times 10^{-6}=69.1-7.4{\left(\frac{t^{3}}{d^{3}}\right)}^{-0.5}\times 10^{-6}$$

By solving Equation (21) with MATLAB_R2019b software (MathWorks, Natick, MA, USA), the optimized value of *n*_p_ with different *t/d* can be calculated, as shown in Table 4. Then, the optimized constitutive model can be written as:

(22) $$\sigma_{t}=(832.7+102.3\sqrt{t/d}+30.6{\left(t/d\right)}^{-1}){\left(0.0043+\varepsilon_{t}\right)}^{n_{p}}.$$

**Figure 9:**
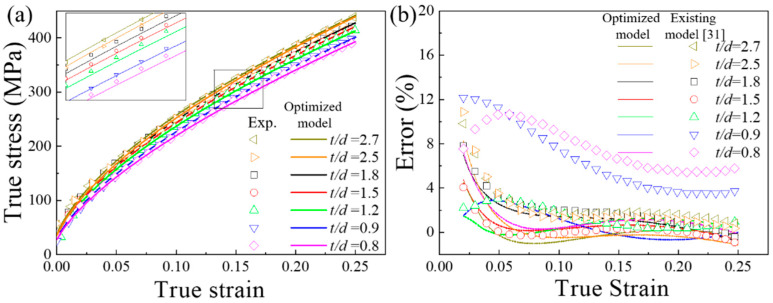
(**a**) Prediction of true stress-strain curves for the optimized constitutive model (**b**) Comparison of error between the optimized constitutive model and the existing constitutive model.

The prediction of the optimized existing model is shown in Figure 9a. Compared to the large strain conditions (*ε* > 0.05), both constitutive models have larger errors for the small strain conditions (*ε* < 0.05). The error between the optimized constitutive model and the experimental result and the error between the prediction result of the existing constitutive model and the experimental result are shown in Figure 9b. The prediction accuracy of the two models for specimens with a *t/d* > 1 was high. However, for *t/d* < 1 specimens, the optimized model expresses significantly lower errors in predicting results than existing models. The prediction accuracy of the optimized constitutive equation for specimens with a *t/d* < 1 was significantly improved. For specimens with a *t/d* > 1, the prediction results of the optimized constitute model also excellently conformed with the experimental results. Avoiding the effect of considering microstructure deformation mechanisms is an advantage of new models that include energy inputs to improve prediction. The new model based on the Energy Conservation Law successfully optimized the strain hardening strength *n* in the existing constitutive model based on the Swift equation.

## 4. Conclusions

The new model based on the Energy Conservation Law was established to further characterize the size effect on flow stress. In this paper, the energy conversion during plastic deformation is analyzed. The results show that for cold metal forming with little friction, the total work done by the experimental machine on the specimen is almost equal to the energy consumed by the plastic deformation based on the Energy Conservation Law. Taking the uniaxial tensile test as an example, the total work done by the experimental machine on the specimen can be calculated from the force-displacement curve and the energy consumed by the plastic deformation can be calculated from the true stress-strain curve. Based on the Energy Conservation Law, the analysis shows that the true stress-strain curve reflects the average stress state of the grains per unit volume. The new model can be used to optimize the parameters of the flow stress constitutive equation about the size effect. The existing model significantly improved the prediction accuracy after the optimization of the new model involving energy.

## Figures and Tables

**Table 1:** Material parameters for the Swift model.

Test	*t* (μm)	Annealing Temperature (°C)	*d* (μm)	*t/d*	*K*(MPa)	*n*	*ε*_0_
1	100	750	55.2	1.8	958.8	0.59	0.0043
2	100	850	66.7	1.5	968.2	0.61	0.0043
3	100	950	81.5	1.2	987.5	0.63	0.0043
4	100	1050	109.5	0.9	998.8	0.66	0.0043
5	100	1150	124.9	0.8	958.2	0.65	0.0043
6	200	750	73.0	2.7	1044.1	0.64	0.0043
7	200	850	81.0	2.5	1030.6	0.65	0.0043

**Table 2:** The initial and modified values for *n* and *K*(*t/d*).

Annealed	*n*1	*n*2	*n*6	*K*1(*t/d*)/(MPa)	*K*2(*t/d*)/(MPa)	*K*6(*t/d*)/(MPa)	*ε*_0_
Initial	0.59	0.61	0.64	958.8	968.2	1044.1	0.0043
Modified	*n* = (*n*1 + *n*2 + *n*6)/3 = 0.61			988.5	978.4	1013.3	0.0043

**Table 3:** $\bar{E}$ of specimens with various *t/d.*

Test	1	2	3	4	5	6	7
*t/d*	1.8	1.5	1.2	0.9	0.8	2.7	2.5
$\bar{E}$ (10^−6^ J)	66.0	64.9	63.8	60.7	59.7	67.8	67.1

**Table 4:** Optimized *n*_p_ value based on the new model.

Test	1	2	3	4	5	6	7
*t/d*	1.8	1.5	1.2	0.9	0.8	2.7	2.5
*n*_p_	0.61	0.62	0.63	0.64	0.66	0.60	0.61

## References

[1] Lai X., Peng L., Hu P., Ni J. (2008). Material behavior modelling in micro/meso-scale forming process with considering size/scale effects. Comp. Mater. Sci..

[2] Meng B., Wang W.H., Zhang Y.Y., Wan M. (2019). Size effect on plastic anisotropy in microscale deformation of metal foil. J. Mater. Process. Tech..

[3] Gu R., Ngan A.H.W. (2013). Size effect on the deformation behavior of duralumin micropillars. Scripta. Mater..

[4] Geiger M., Vollertsen F., Kals R. (1996). Fundamentals on the manufacturing of sheet metal microparts. Annal. CIRP.

[5] Engel U., Eckstein R. (2002). Microfilming-from basic research to its realization. J. Mater. Process. Tech..

[6] Leu D.K. (2015). Distinguishing micro-scale from macro-scale tensile flow stress of sheet metals in microforming. Mater. Des..

[7] Kim G.Y., Ni J., Koc M. (2007). Modeling of the size effects on the behavior of metals in microscale deformation processes. ASME J. Manuf. Sci. Eng..

[8] Keller C., Hug E. (2008). Hall-Petch behaviour of Ni polycrystals with a few grains per thickness. Mater. Lett..

[9] Keller C., Hug E., Retoux R., Feaugas X. (2010). TEM study of dislocation patterns in near-surface and core regions of deformed nickel polycrystals with few grains across the cross section. Mech. Mater..

[10] Janssen P.J.M., Keijser T.H.D., Geers M.G.D. (2006). An experimental assessment of grain size effects in the uniaxial straining of thin Al sheet with a few grains across the thickness. Mater. Sci. Eng. A..

[11] Hall E.O. (1951). The Deformation and Aging of Mild Steel. Proc. Phys. Soc. Lond. Sect B.

[12] Petch N.J. (1953). The Cleavage Strength of Polycrystals. J. Iron Steel Inst. Lond..

[13] Meng B., Fu M.W. (2015). Size effect on deformation behavior and ductile fracture in microforming of pure copper sheets considering free surface roughening. Mater. Des..

[14] Furushima T., Tsunezaki H., Manabe K.I., Alexsandrov S. (2014). Ductile fracture and free surface roughening behaviors of pure copper foils for micro/meso-scale forming. Int. J. Mach. Tool. Manu..

[15] Kim H.S., Lee Y.S. (2012). Size dependence of flow stress and plastic behaviour in microforming of polycrystalline metallic materials. Proc. Inst. Mech. Eng. Part C J. Eng. Mech. Eng. Sci..

[16] Wang C.J., Xue S.X., Chen G., Zhang P. (2017). Constitutive model based on dislocation density and ductile fracture of Monel 400 thin sheet under tension. Met. Mater. Int..

[17] Lyu H., Ruimi A., Zbib H.M. (2015). A dislocation-based model for deformation and size effect in multi-phase steels. Int. J. Plast..

[18] Miyazaki S., Shibata K., Fujita H. (1979). Effect of specimen thickness on mechanical properties of polycrystalline aggregates with various grain sizes. Acta. Metall..

[19] Meyersm M.A., Ashworth E. (1982). A model for the effect of grain size on the yield stress of metals. Philos. Mag. A..

[20] Geiger M., Geißdörfer S., Engel U. (2007). Mesoscopic model: Advanced simulation of microforming processes. Prod. Eng..

[21] Molotnikov A., Lapovok R., Davies C.H.J., Cao W., Estrinabc Y. (2008). Size effect on the tensile strength of fine-grained copper. Scripta. Mater..

[22] Liu J.G., Fu M.W., Chan W.L. (2012). A constitutive model for modeling of the deformation behavior in microforming with a consideration of grain boundary strengthening. Comp. Mater. Sci..

[23] Keller C., Hug E., Feaugas X. (2011). Microstructural size effects on mechanical properties of high purity nickel. Int. J. Plasticity..

[24] Vollertsen F. (2012). Effects on the deep drawing diagram in micro forming. Prod. Eng..

[25] Hollomon J.H. (1945). Tensile deformation. Trans. AIME.

[26] Mahabunphachai S., Koç M. (2008). Investigation of size effects on material behavior of thin sheet metals using hydraulic bulge testing at micro/meso-scales. J. Mach. Tools. Manuf..

[27] Starink M.J. (2017). Dislocation versus grain boundary strengthening in SPD processed metals: Non-causal relation between grain size and strength of deformed polycrystals. Mater. Sci. Eng. A..

[28] Cordero N.M., Forest S., Busso E.P., Berbenni S., Cherkaouic M. (2012). Grain size effects on plastic strain and dislocation density tensor fields in metal polycrystals. Comp. Mater. Sci..

[29] Chan W.L., Fu M.W. (2011). Experimental studies and numerical modeling of the specimen and grain size effects on the flow stress of sheet metal in microforming. Mater. Sci. Eng. A..

[30] Evers L.P., Brekelmans W.A.M., Geers M.G.D. (2004). Scale dependent crystal plasticity framework with dislocation density and grain boundary effects. Int. J. Solids Struct..

[31] Leu D.-K. (2009). Modeling of Size Effect on Tensile Flow Stress of Sheet Metal in Microforming. J. Manuf. Sci. Eng..

[32] Lederer M., Gröger V., Khatibi G., Weiss B. (2010). Size dependency of mechanical properties of high purity aluminium foils. Mater. Sci. Eng. A.

[33] Liu M.Q., Liu Y.L., Yan Y., Han D., Li X.W. (2017). Thickness-dependent tensile and fatigue behavior of a single-slip-oriented Cu single crystal. Cryst. Res. Techmol..

[34] Peng L., Xu Z., Gao Z., Fu M.W. (2018). A constitutive model for metal plastic deformation at micro/meso scale with consideration of grain orientation and its evolution. Int. J. Mech. Sci..

[35] Wang Y., Dong P.L., Xu Z.Y., Yan H., Wang J.M., Wang J.J. (2010). A constitutive model for thin sheet metal in micro-forming considering first order size effects. Mater. Des..

